# Supplementary material for: Modeling glioblastoma heterogeneity as a dynamic network of cell states
Source: Mol Syst Biol. 2021 Sep 16;17(9):e10105. doi: 10.15252/msb.202010105 (PMC8444284; doi:10.15252/msb.202010105)
Supplement: Supplementary file 6 — Source Data for Figure 5 [file MSB-17-e10105-s004.zip › Figure5A_sourcedata/GSEA_3071/nosigdb_stateII.GseaPreranked.1622795151171/DOROSZKO_U3013MG_CLUSTER_2.html]

Details for gene set DOROSZKO\_U3013MG\_CLUSTER\_2[GSEA]

|  || Dataset | state23071 |
| Phenotype | NoPhenotypeAvailable |
| Upregulated in class | na\_pos |
| GeneSet | DOROSZKO\_U3013MG\_CLUSTER\_2 |
| Enrichment Score (ES) | 0.7843977 |
| Normalized Enrichment Score (NES) | 5.1675005 |
| Nominal p-value | 0.0 |
| FDR q-value | 0.0 |
| FWER p-Value | 0.0 |
Table: GSEA Results Summary

  

Fig 1: Enrichment plot: DOROSZKO\_U3013MG\_CLUSTER\_2      
 Profile of the Running ES Score & Positions of GeneSet Members on the Rank Ordered List

  

| PROBE | GENE SYMBOL | GENE\_TITLE | RANK IN GENE LIST | RANK METRIC SCORE | RUNNING ES | CORE ENRICHMENT || 1 | CLSPN |  |  | 1 | 1.984 | 0.0134 | Yes |
| 2 | HIST1H4C |  |  | 2 | 1.902 | 0.0264 | Yes |
| 3 | RRM2 |  |  | 3 | 1.857 | 0.0391 | Yes |
| 4 | ANLN |  |  | 4 | 1.760 | 0.0512 | Yes |
| 5 | TYMS |  |  | 8 | 1.665 | 0.0619 | Yes |
| 6 | PCLAF |  |  | 10 | 1.638 | 0.0729 | Yes |
| 7 | CDK1 |  |  | 14 | 1.540 | 0.0827 | Yes |
| 8 | DIAPH3 |  |  | 15 | 1.531 | 0.0932 | Yes |
| 9 | ZWINT |  |  | 16 | 1.510 | 0.1035 | Yes |
| 10 | HJURP |  |  | 17 | 1.490 | 0.1137 | Yes |
| 11 | ATAD5 |  |  | 18 | 1.469 | 0.1238 | Yes |
| 12 | FEN1 |  |  | 19 | 1.464 | 0.1339 | Yes |
| 13 | UBE2C |  |  | 23 | 1.438 | 0.1430 | Yes |
| 14 | CENPU |  |  | 24 | 1.416 | 0.1527 | Yes |
| 15 | RAD51AP1 |  |  | 25 | 1.384 | 0.1622 | Yes |
| 16 | CENPK |  |  | 26 | 1.382 | 0.1717 | Yes |
| 17 | MYBL2 |  |  | 28 | 1.362 | 0.1807 | Yes |
| 18 | USP1 |  |  | 31 | 1.357 | 0.1895 | Yes |
| 19 | PSMC3IP |  |  | 36 | 1.322 | 0.1976 | Yes |
| 20 | RRM1 |  |  | 37 | 1.310 | 0.2066 | Yes |
| 21 | ORC6 |  |  | 39 | 1.308 | 0.2153 | Yes |
| 22 | CDCA5 |  |  | 41 | 1.289 | 0.2239 | Yes |
| 23 | TK1 |  |  | 42 | 1.281 | 0.2327 | Yes |
| 24 | AURKB |  |  | 44 | 1.276 | 0.2412 | Yes |
| 25 | BRCA1 |  |  | 49 | 1.249 | 0.2487 | Yes |
| 26 | CCNA2 |  |  | 53 | 1.230 | 0.2564 | Yes |
| 27 | DNAJC9 |  |  | 56 | 1.217 | 0.2643 | Yes |
| 28 | SKA3 |  |  | 57 | 1.215 | 0.2726 | Yes |
| 29 | MAD2L1 |  |  | 61 | 1.199 | 0.2801 | Yes |
| 30 | UBE2T |  |  | 62 | 1.192 | 0.2882 | Yes |
| 31 | ATAD2 |  |  | 63 | 1.189 | 0.2964 | Yes |
| 32 | RFC3 |  |  | 67 | 1.157 | 0.3036 | Yes |
| 33 | SPC25 |  |  | 68 | 1.156 | 0.3115 | Yes |
| 34 | MELK |  |  | 69 | 1.145 | 0.3193 | Yes |
| 35 | TOP2A |  |  | 72 | 1.132 | 0.3266 | Yes |
| 36 | KIF2C |  |  | 73 | 1.132 | 0.3344 | Yes |
| 37 | FAM111A |  |  | 80 | 1.112 | 0.3405 | Yes |
| 38 | GMNN |  |  | 83 | 1.108 | 0.3476 | Yes |
| 39 | CEP55 |  |  | 87 | 1.096 | 0.3543 | Yes |
| 40 | KIFC1 |  |  | 88 | 1.094 | 0.3618 | Yes |
| 41 | NDC80 |  |  | 94 | 1.074 | 0.3679 | Yes |
| 42 | MASTL |  |  | 96 | 1.063 | 0.3750 | Yes |
| 43 | TMPO |  |  | 99 | 1.056 | 0.3817 | Yes |
| 44 | NCAPG |  |  | 100 | 1.056 | 0.3890 | Yes |
| 45 | DHFR |  |  | 101 | 1.051 | 0.3962 | Yes |
| 46 | MND1 |  |  | 104 | 1.036 | 0.4028 | Yes |
| 47 | MKI67 |  |  | 107 | 1.031 | 0.4093 | Yes |
| 48 | TPX2 |  |  | 111 | 1.025 | 0.4156 | Yes |
| 49 | CDCA8 |  |  | 113 | 1.018 | 0.4223 | Yes |
| 50 | TRIP13 |  |  | 114 | 1.014 | 0.4293 | Yes |
| 51 | KIF11 |  |  | 115 | 1.014 | 0.4362 | Yes |
| 52 | NCAPH |  |  | 116 | 1.013 | 0.4432 | Yes |
| 53 | NCAPD3 |  |  | 117 | 1.013 | 0.4501 | Yes |
| 54 | ASF1B |  |  | 120 | 1.007 | 0.4565 | Yes |
| 55 | SMC2 |  |  | 123 | 1.005 | 0.4629 | Yes |
| 56 | NUF2 |  |  | 125 | 1.004 | 0.4696 | Yes |
| 57 | DEPDC1 |  |  | 126 | 0.999 | 0.4764 | Yes |
| 58 | CKS1B |  |  | 128 | 0.999 | 0.4830 | Yes |
| 59 | TACC3 |  |  | 130 | 0.992 | 0.4896 | Yes |
| 60 | KIF20B |  |  | 133 | 0.989 | 0.4958 | Yes |
| 61 | FANCI |  |  | 138 | 0.984 | 0.5016 | Yes |
| 62 | ESCO2 |  |  | 144 | 0.971 | 0.5070 | Yes |
| 63 | CKAP2L |  |  | 148 | 0.959 | 0.5128 | Yes |
| 64 | KIF23 |  |  | 161 | 0.936 | 0.5161 | Yes |
| 65 | SHCBP1 |  |  | 164 | 0.933 | 0.5220 | Yes |
| 66 | RTKN2 |  |  | 166 | 0.931 | 0.5282 | Yes |
| 67 | GTSE1 |  |  | 167 | 0.929 | 0.5345 | Yes |
| 68 | CDCA2 |  |  | 171 | 0.922 | 0.5401 | Yes |
| 69 | CENPM |  |  | 178 | 0.901 | 0.5448 | Yes |
| 70 | SAC3D1 |  |  | 188 | 0.887 | 0.5486 | Yes |
| 71 | FAM83D |  |  | 197 | 0.876 | 0.5525 | Yes |
| 72 | SPDL1 |  |  | 199 | 0.874 | 0.5583 | Yes |
| 73 | C21orf58 |  |  | 201 | 0.873 | 0.5640 | Yes |
| 74 | PBK |  |  | 214 | 0.864 | 0.5669 | Yes |
| 75 | CDCA3 |  |  | 219 | 0.858 | 0.5717 | Yes |
| 76 | CENPH |  |  | 221 | 0.855 | 0.5774 | Yes |
| 77 | BUB1B |  |  | 223 | 0.851 | 0.5829 | Yes |
| 78 | ASPM |  |  | 231 | 0.842 | 0.5869 | Yes |
| 79 | EZH2 |  |  | 233 | 0.839 | 0.5924 | Yes |
| 80 | DBF4 |  |  | 240 | 0.832 | 0.5966 | Yes |
| 81 | RACGAP1 |  |  | 241 | 0.831 | 0.6023 | Yes |
| 82 | PRC1 |  |  | 242 | 0.830 | 0.6080 | Yes |
| 83 | LMNB1 |  |  | 251 | 0.824 | 0.6116 | Yes |
| 84 | TUBB4B |  |  | 271 | 0.784 | 0.6122 | Yes |
| 85 | YWHAH |  |  | 273 | 0.781 | 0.6173 | Yes |
| 86 | HMGB2 |  |  | 276 | 0.780 | 0.6221 | Yes |
| 87 | TUBA1C |  |  | 277 | 0.779 | 0.6275 | Yes |
| 88 | SMC4 |  |  | 279 | 0.777 | 0.6325 | Yes |
| 89 | CENPE |  |  | 284 | 0.775 | 0.6368 | Yes |
| 90 | SMTN |  |  | 292 | 0.766 | 0.6403 | Yes |
| 91 | CENPA |  |  | 303 | 0.751 | 0.6429 | Yes |
| 92 | NSD2 |  |  | 308 | 0.747 | 0.6470 | Yes |
| 93 | KIF4A |  |  | 309 | 0.746 | 0.6521 | Yes |
| 94 | CKS2 |  |  | 312 | 0.745 | 0.6568 | Yes |
| 95 | PRR11 |  |  | 314 | 0.743 | 0.6616 | Yes |
| 96 | ALYREF |  |  | 315 | 0.741 | 0.6667 | Yes |
| 97 | CENPN |  |  | 320 | 0.737 | 0.6707 | Yes |
| 98 | ANP32B |  |  | 325 | 0.734 | 0.6747 | Yes |
| 99 | CENPW |  |  | 330 | 0.727 | 0.6787 | Yes |
| 100 | SPC24 |  |  | 340 | 0.719 | 0.6813 | Yes |
| 101 | ARHGAP11A |  |  | 346 | 0.712 | 0.6850 | Yes |
| 102 | LMNB2 |  |  | 352 | 0.709 | 0.6885 | Yes |
| 103 | AURKA |  |  | 360 | 0.705 | 0.6916 | Yes |
| 104 | DTYMK |  |  | 364 | 0.701 | 0.6956 | Yes |
| 105 | TTK |  |  | 365 | 0.701 | 0.7005 | Yes |
| 106 | KIF14 |  |  | 378 | 0.693 | 0.7022 | Yes |
| 107 | PARPBP |  |  | 382 | 0.688 | 0.7061 | Yes |
| 108 | MT2A |  |  | 393 | 0.682 | 0.7082 | Yes |
| 109 | KIF15 |  |  | 404 | 0.676 | 0.7103 | Yes |
| 110 | CCDC34 |  |  | 411 | 0.672 | 0.7134 | Yes |
| 111 | TEDC1 |  |  | 414 | 0.670 | 0.7175 | Yes |
| 112 | SGO2 |  |  | 415 | 0.670 | 0.7221 | Yes |
| 113 | KPNA2 |  |  | 420 | 0.669 | 0.7257 | Yes |
| 114 | FOXM1 |  |  | 423 | 0.667 | 0.7297 | Yes |
| 115 | ZWILCH |  |  | 436 | 0.659 | 0.7312 | Yes |
| 116 | BUB3 |  |  | 441 | 0.657 | 0.7347 | Yes |
| 117 | NCAPD2 |  |  | 457 | 0.647 | 0.7353 | Yes |
| 118 | HMMR |  |  | 475 | 0.639 | 0.7354 | Yes |
| 119 | CKLF |  |  | 482 | 0.637 | 0.7382 | Yes |
| 120 | PGP |  |  | 492 | 0.631 | 0.7403 | Yes |
| 121 | RHEB |  |  | 500 | 0.629 | 0.7428 | Yes |
| 122 | POP7 |  |  | 502 | 0.628 | 0.7469 | Yes |
| 123 | KNL1 |  |  | 504 | 0.628 | 0.7509 | Yes |
| 124 | BIRC5 |  |  | 505 | 0.628 | 0.7552 | Yes |
| 125 | ECT2 |  |  | 510 | 0.626 | 0.7585 | Yes |
| 126 | SGO1 |  |  | 528 | 0.612 | 0.7584 | Yes |
| 127 | RPA3 |  |  | 532 | 0.611 | 0.7618 | Yes |
| 128 | BTG3 |  |  | 540 | 0.607 | 0.7642 | Yes |
| 129 | DDX39A |  |  | 542 | 0.605 | 0.7681 | Yes |
| 130 | CENPQ |  |  | 569 | 0.590 | 0.7655 | Yes |
| 131 | MIS18BP1 |  |  | 574 | 0.588 | 0.7685 | Yes |
| 132 | CENPF |  |  | 575 | 0.588 | 0.7726 | Yes |
| 133 | CCNB1 |  |  | 579 | 0.587 | 0.7758 | Yes |
| 134 | EXOSC8 |  |  | 604 | 0.576 | 0.7737 | Yes |
| 135 | CACYBP |  |  | 613 | 0.573 | 0.7756 | Yes |
| 136 | CKAP2 |  |  | 619 | 0.569 | 0.7782 | Yes |
| 137 | SPAG5 |  |  | 642 | 0.555 | 0.7764 | Yes |
| 138 | NUSAP1 |  |  | 654 | 0.550 | 0.7774 | Yes |
| 139 | H2AFX |  |  | 671 | 0.544 | 0.7770 | Yes |
| 140 | MNS1 |  |  | 672 | 0.543 | 0.7808 | Yes |
| 141 | WEE1 |  |  | 685 | 0.536 | 0.7814 | Yes |
| 142 | CIP2A |  |  | 696 | 0.533 | 0.7825 | Yes |
| 143 | DLGAP5 |  |  | 712 | 0.529 | 0.7823 | Yes |
| 144 | GPSM2 |  |  | 719 | 0.526 | 0.7844 | Yes |
| 145 | PHF19 |  |  | 745 | 0.518 | 0.7816 | No |
| 146 | PTGES3 |  |  | 763 | 0.512 | 0.7808 | No |
| 147 | CDC20 |  |  | 844 | 0.486 | 0.7637 | No |
| 148 | CDKN2D |  |  | 911 | 0.468 | 0.7502 | No |
| 149 | ITGB3BP |  |  | 935 | 0.462 | 0.7475 | No |
| 150 | HIRIP3 |  |  | 936 | 0.462 | 0.7506 | No |
| 151 | KIF22 |  |  | 975 | 0.450 | 0.7441 | No |
| 152 | MAGOH |  |  | 1007 | 0.443 | 0.7392 | No |
| 153 | PLK1 |  |  | 1009 | 0.442 | 0.7420 | No |
| 154 | POC1A |  |  | 1080 | 0.421 | 0.7271 | No |
| 155 | NEK2 |  |  | 1100 | 0.417 | 0.7251 | No |
| 156 | CBX5 |  |  | 1132 | 0.412 | 0.7200 | No |
| 157 | BUB1 |  |  | 1145 | 0.408 | 0.7198 | No |
| 158 | APOLD1 |  |  | 1239 | 0.389 | 0.6988 | No |
| 159 | BCL2L12 |  |  | 1252 | 0.386 | 0.6984 | No |
| 160 | ANP32E |  |  | 1334 | 0.371 | 0.6803 | No |
| 161 | KNSTRN |  |  | 1458 | 0.346 | 0.6514 | No |
| 162 | CBR3 |  |  | 1522 | 0.336 | 0.6376 | No |
| 163 | CDC27 |  |  | 1562 | 0.330 | 0.6300 | No |
| 164 | REEP4 |  |  | 1786 | 0.300 | 0.5753 | No |
| 165 | OIP5 |  |  | 1789 | 0.299 | 0.5768 | No |
| 166 | ILF2 |  |  | 1833 | 0.294 | 0.5679 | No |
| 167 | SKA2 |  |  | 2061 | 0.266 | 0.5119 | No |
| 168 | CKAP5 |  |  | 2135 | 0.258 | 0.4951 | No |
| 169 | MXD3 |  |  | 2156 | 0.256 | 0.4918 | No |
| 170 | SOGA1 |  |  | 2731 | -0.322 | 0.3479 | No |
Table: GSEA details [plain text format]

  

Fig 2: DOROSZKO\_U3013MG\_CLUSTER\_2: Random ES distribution      
 Gene set null distribution of ES for **DOROSZKO\_U3013MG\_CLUSTER\_2**
